# Nomograms for Predicting Axillary Lymph Node Status Reconciled With Preoperative Breast Ultrasound Images

**DOI:** 10.3389/fonc.2021.567648

**Published:** 2021-04-07

**Authors:** Dongmei Liu, Yujia Lan, Lei Zhang, Tong Wu, Hao Cui, Ziyao Li, Ping Sun, Peng Tian, Jiawei Tian, Xia Li

**Affiliations:** ^1^ Department of Ultrasound, The Second Affiliated Hospital, Harbin, China; ^2^ College of Bioinformatics Science and Technology, Harbin Medical University, Harbin, China

**Keywords:** nomogram, breast cancer, ultrasound image, axillary lymph node, predicting

## Abstract

**Introduction:**

The axillary lymph node (ALN) status of breast cancer patients is an important prognostic indicator. The use of primary breast mass features for the prediction of ALN status is rare. Two nomograms based on preoperative ultrasound (US) images of breast tumors and ALNs were developed for the prediction of ALN status.

**Methods:**

A total of 743 breast cancer cases collected from 2016 to 2019 at the Second Affiliated Hospital of Harbin Medical University were randomly divided into a training set (n = 523) and a test set (n = 220). A primary tumor feature model (PTFM) and ALN feature model (ALNFM) were separately generated based on tumor features alone, and a combination of features was used for the prediction of ALN status. Logistic regression analysis was used to construct the nomograms. A receiver operating characteristic curve was plotted to obtain the area under the curve (AUC) to evaluate accuracy, and bias-corrected AUC values and calibration curves were obtained by bootstrap resampling for internal and external verification. Decision curve analysis was applied to assess the clinical utility of the models.

**Results:**

The AUCs of the PTFM were 0.69 and 0.67 for the training and test sets, respectively, and the bias-corrected AUCs of the PTFM were 0.67 and 0.67, respectively. Moreover, the AUCs of the ALNFM were 0.86 and 0.84, respectively, and the bias-corrected AUCs were 0.85 and 0.81, respectively. Compared with the PTFM, the ALNFM showed significantly improved prediction accuracy (*p* < 0.001). Both the calibration and decision curves of the ALNFM nomogram indicated greater accuracy and clinical practicality. When the US tumor size was ≤21.5 mm, the Spe was 0.96 and 0.92 in the training and test sets, respectively. When the US tumor size was greater than 21.5 mm, the Sen was 0.85 in the training set and 0.87 in the test set. Our further research showed that when the US tumor size was larger than 35 mm, the Sen was 0.90 in the training set and 0.93 in the test set.

**Conclusion:**

The ALNFM could effectively predict ALN status based on US images especially for different US tumor size.

## Introduction

Axillary lymph node (ALN) status has been considered to be the most important prognostic indicator for breast cancer and the main determinant of adjuvant systemic therapy (1, 2). However, the role of ALN status in the treatment of breast cancer is still a controversial topic. In addition, breast surgery tends to be less invasive and painless (3). ALN dissection (ALND), sentinel lymph node biopsy (SLNB) and axillary sampling are currently used for pathological examination of the ALNs. Although less commonly reported, secondary injury involving anaphylaxis, infection, hematoma, seroma and paresthesia could lead to a prolonged surgical time and financial burden to the patients (4); even axillary biopsy is an invasive procedure. With the increasing detection of small-size or early-stage breast cancer, the incidence of lymph node metastasis has decreased worldwide. Previous studies showed that approximately 65% to 75% of sentinel lymph nodes (SLNs) are negative; thus, SLNB may not be necessary for those patients, which has become a controversial issue (5, 6).

In recent years, medical researchers have attempted to explore some noninvasive methods for predicting SLN status preoperatively to avoid unnecessary ALND, SLNB or lymph node puncture biopsy. The diagnostic value of mammograms and computed tomography (CT) for predicting ALN status is not high (7). Both magnetic resonance imaging (MRI) and positron emission tomography-computed tomography (PET-CT) are not common imaging methods for breast lesions because of their high cost and lack of universal coverage (8, 9). Therefore, ultrasound (US) is widely used in the preoperative examination of breast cancer lesions. With the development of US technology, the strategy of ALN management has been further improved. Preoperatively, determining the ALN status can improve personal and multidisciplinary treatment plans. Currently, there is no unified diagnostic standard for US detection of ALN status. A large number of clinical trials have confirmed that although ALNs cannot be found by US, ALN metastasis may be present (10, 11). In several studies, scholars applied preoperative and postoperative data to establish an SLN metastasis prediction model, and the performance could reach 0.70 to 0.79 (12–14). The prediction effect was not ideal, especially for postoperative prediction, with little clinical application value.

This research aimed to explore the preoperative sonographic features of ALNs and breast masses and clinicopathological features related to ALN status to build two nomograms for predicting ALN status. These models were expected to have better accuracy than the previous methods, provide a more valuable reference for clinical treatment, and make it possible to avoid invasive axillary surgery (ALND, SLNB or lymph node biopsy) to minimize trauma.

## Methods

**Figure 1:**
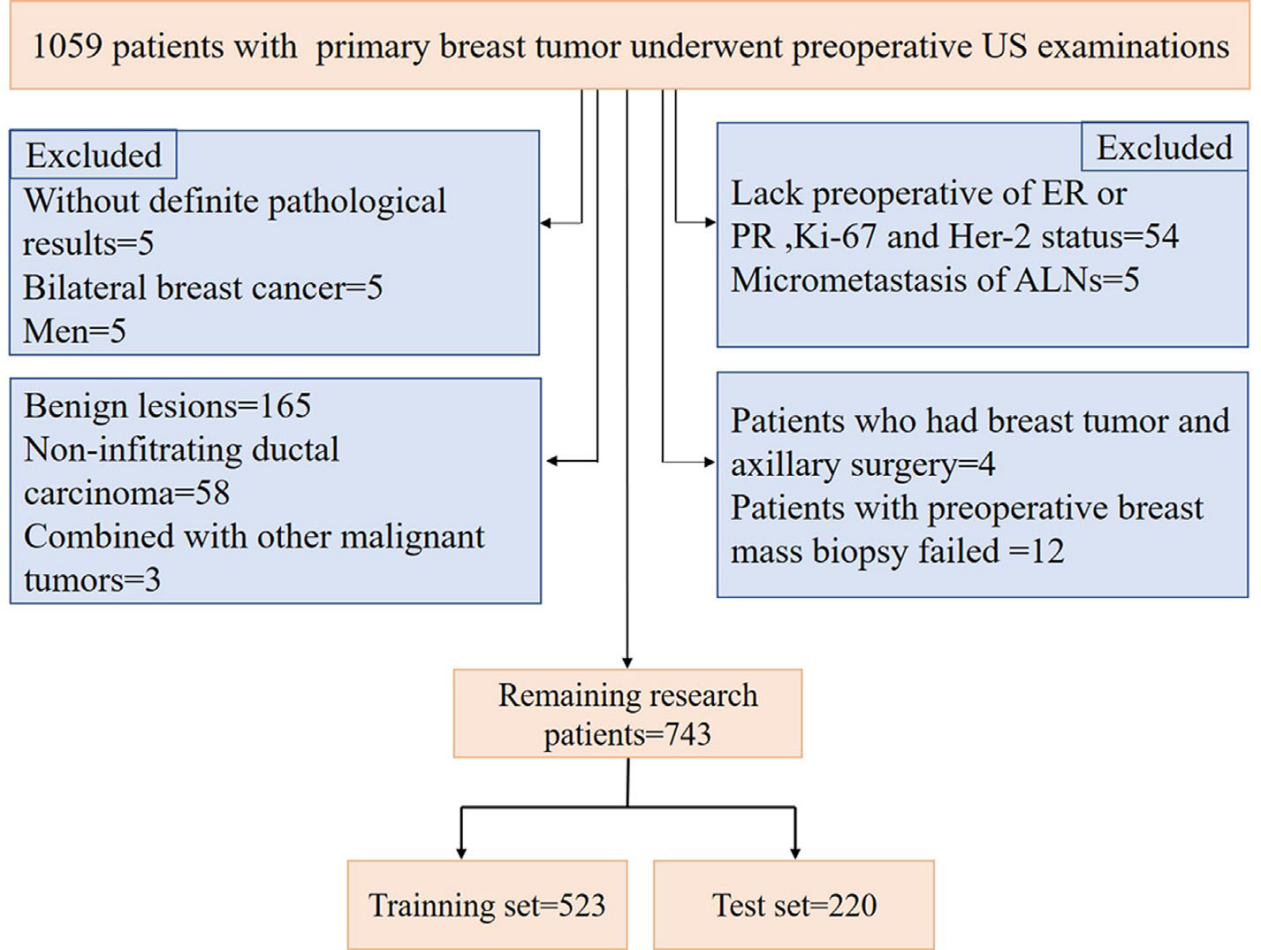
Recruitment process for study patients.

A total of 1059 patients with breast tumors were collected from the Second Affiliated Hospital of Harbin Medical University from Nov. 10, 2016, to Dec. 30, 2019. For breast masses that were suspected to be malignant preoperatively, a preoperative core needle biopsy of the breast masses and suspicious ALNs could be performed first. Neoadjuvant therapy (neoadjuvant chemotherapy, targeted therapy and endocrine therapy) could be used for patients with large masses, those who needed breast conservative surgery, and those with local late-stage disease and stage II disease with poor molecular biological characteristics. For patients with operable masses, a comprehensive surgical treatment (modified radical mastectomy, mastectomy and SLNB, breast conservative surgery, and reconstruction) could be selected based on the patient’s condition. Postoperative radiotherapy, chemotherapy, targeted therapy, endocrine therapy, immunotherapy, and traditional Chinese medicine treatment were selected according to the patient’s condition. The procedure used for patient selection is shown in **Figure 1**. The inclusion criteria were as follows: invasive breast cancer patients with complete clinical information and US images, patients with preoperative breast mass core needle biopsy, and those with ALNs with SLNB, ALND or core needle biopsy, the patients selected in our study were all before neoadjuvant therapy or not received neoadjuvant therapy. The exclusion criteria were as follows: benign lesions, noninvasive carcinoma, and presence of other malignant tumors. According to the above criteria, 743 patients were finally selected for model construction. US examinations were performed by a board-certified sonographer with 8 years of experience in breast US imaging using a Supersonic Imagine Aixplorer (France) coupled with linear probe arrays of 10 to 2 MHz and 15 to 4 MHz. To maintain consistency, after scanning the entire breast and ipsilateral ALN using B-mode US and color Doppler flow imaging (CDFI), the sonographer selected images of the target tumor and ALN in sagittal and transverse planes and stored dynamic and static images for a double-blind analysis.

The expression levels of the estrogen receptor (ER) and progesterone receptor (PR, positive, >1% of cells staining positive) (15), human epidermal growth factor receptor 2 (HER-2/neu, positive, score of 3+ on immunohistochemistry, score of 2+ on fluorescence *in situ* hybridization) (16), and Ki-67 index (threshold over 14%) (17) were determined by immunohistochemical staining by applying routine methods after preoperative core needle biopsy. Core needle biopsy or mastectomy was used to determine breast pathology. SLNB, ALND, or core needle biopsy was used to determine macrometastasis of the ALNs.

### Analysis of US Features

Gray-scale US features were evaluated according to the uniform American College of Radiology Breast Imaging-Reporting and Data System (ACR-BI-RADS) (18), and the color Doppler flow grade was determined according to the Alder classification criteria (19). To ensure consistency of the image analysis results, two sonographers with 10 and 12 years of clinical experience with breast US were responsible for independent retrospective analysis of the images. In cases of disagreement, a consensus was reached. Because there is no unified diagnostic standard for ALN ultrasound, some high-level related literature was consulted (20–22). All features are shown in **Table 1**. Based on the above features, we developed the following two models: the primary tumor feature model (PTFM) was established using clinical features and US features of primary breast tumors, and the ALN features model (ALNFM) was developed by considering clinical features and US features of primary breast tumors and US features of ALNs. The optimal model was screened out and extensively studied according to tumor size.

**Table 1:** Patients: all patients, patients according to training set or test set and comparison of the composition ratio between groups.

Features	All patients, n(%)	Training set, n(%)	Test set, n(%)	*P* value
Age(y)	48.00(23.00,88.00)	47.00(23.00,88.00)	51.58±10.30	0.097
Quantity				0.639
Single	617(83.04)	437(83.56)	180(81.82)	
Multiple	126(16.95)	86(16.44)	40(18.18)	
Shape				0.971
Oval, round	89(11.98)	62(11.85)	27(12.27)	
Irregular	654(88.02)	461(88.15)	193(87.73)	
Orientation				1.000
Parallel	473(63.66)	333(63.67)	140(63.64)	
Not parallel	270(36.34)	190(36.33)	80(36.36)	
Margin contour				0.974
Smooth	52(7.00)	36(6.88)	16(7.27)	
Lobulate	120(16.15)	84(16.06)	36(16.36)	
Angular, Spiculate	571(76.85)	403(77.06)	168(76.37)	
Margin				0.980
Circumscribed	56(7.54)	40(7.54)	16(7.27)	
Not circumscribed	687(92.46)	483(92.46)	204(92.73)	
Boundary				0.728
Abrupt interface	367(49.39)	261(49.90)	106(48.18)	
Echogenic halo	376(50.61)	262(50.10)	114(51.12)	
Main posterior echo				0.868
Shadowing	317(42.66)	220(42.07)	97(44.09)	
Indifferent	413(55.59)	294(56.21)	119(54.09)	
Enhancement	13(1.75)	9(1.72)	4(1.82)	
Microcalcification				0.799
Preserved	409(55.05)	295(56.41)	114(51.82)	
Absent	334(44.95)	228(43.59)	106(48.18)	
Echogenicity pattern				0.726
Hypoechoic	717(96.50)	506(96.75)	211(95.91)	
Other echo	26(3.50)	17(3.25)	9(4.09)	
CDFI				0.885
Adler 0,1	274(36.88)	192(36.71)	82(37.27)	
Adler 2,3	469(63.12)	331(63.29)	138(62.73)	
US tumour size(mm)	22,00(5.00,140.00)	22,00(5.00,140.00)	22.00(7.00,75.00)	0.460
ER				0.135
Negative	230(30.96)	171(32.70)	59(26.82)	
Positive	513(69.04)	352(67.30)	161(73.18)	
PR				0.118
Negative	311(41.86)	229(43.79)	82(37.27)	
Positive	432(58.14)	294(56.21)	138(62.73)	
HER-2				0.473
Negative	532(71.60)	379(72.47)	153(69.55)	
Positive	211(28.40)	144(27.53)	67(30.45)	
Ki-67				0.590
Negative	231(31.09)	159(30.40)	72(32.73)	
Positive	512(68.91)	364(69.60)	148(67.27)	
US of lymph node				0.472
No lymph nodes found	311(41.86)	214(40.92)	98(44. 55)	
Lymph nodes found	432(58.14)	309(59.08)	122(55.45)	
Aspect ratio				0.625
No lymph nodes found	318(42.80)	214(40.92)	98(44.55)	
Aspect ratio≥2	151(20.32)	111(21.22)	40(18.18)	
Aspect ratio<2	274(36.88)	198(37.86)	82(37.27)	
Cortical thickness				0.259
No lymph nodes found	311(41.86)	214(40.92)	98(44.55)	
Thickness<3 mm	168(22.61)	118(22.56)	50(22.73)	
Symmetric thickness≥3 mm	27(3.63)	18(3.44)	8(3.64)	
Asymmetric thickness≥3 mm	237(31.90)	173(33.08)	64(29.08)	
Central hilum				0.573
No lymph nodes found	305(41.05)	214(40.92)	98(44.55)	
Preserved	192(25.84)	140(26.77)	45(20.45)	
Partially absent	107(14.40)	70(13.38)	37(16.82)	
Completely absent	139(18.71)	99(18.93)	40(18.18)	
Pathological results of ALNs				0.455
Negative	371(49.93)	256(48.95)	115(52.27)	
Positive	372(50.07)	267(51.05)	105(47.73)	

### Statistical Analysis

A data analysis framework was proposed. Descriptive statistics were applied to assess the frequency distribution among all patients in the study. Interobserver comparisons were evaluated with Cohen’s kappa statistics (23). According to the type of data, the Chi-square test, Mann-Whitney U test and independent t-test were used to compare the two groups (training set vs test set). Univariate logistic regressions were used to evaluate the relationship between each feature and ALN status. Features (*P*<0.05) in the univariate logistic regression analysis that were assessed with the stepwise logistic regression method were used to construct the nomograms. According to the size of the regression coefficient of all independent variables, the scoring standard was established. The risk of predicting ALN status curves and the features value were plotted according to the scales of these scores. Then, a nomogram was developed by plotting a vertical line from the scale of the total score to the scale of the predicted probability.

Using receiver operating characteristic (ROC) curve analysis, the area under the curve (AUC) was obtained, and the Hosmer-Lemeshow (HL) test was applied to assess the fitting accuracy of the final model. The AUC comparison between the two models was performed by DeLong’s test. In addition, ROC curve analysis was used to select the cut-off point of tumor diameter according to ALN status. The bias-corrected AUC and calibration curves were obtained by the bootstrap resampling method and applied for internal and external verification. Decision curve analysis was used to assess the clinical utility of the prediction nomogram. The odds ratio (OR) and 95% confidence interval were used to quantify the association between the features and lymph node status. The conventional statistical analyses were two-sided. Every feature with a *P* value < 0.05 was considered statistically significant, and *P* values were obtained from the Wald test. The statistical software R version 3.5.0, 2.15.3 (http://www.R-project.org/) and Bioconductor packages (http://www.bioconductor.org/) were used for all statistical analyses.

## Results

### General Results

Mastectomy, breast conservative surgery, reconstruction, and neoadjuvant therapy were performed for 75.23% (559/743), 9.29% (69/743), 0.27% (2/743), and 15.21% (113/743) of patients, respectively. In total, 384 and 246 patients received ALND and SLNB, respectively. Preoperative ALN core needle biopsy was carried out in 438 cases; 113 received neoadjuvant therapy, whereas 325 patients did not receive neoadjuvant therapy but underwent ALND or SLNB. A total of 743 patients were randomly divided into two groups: the training set (n=523) and the test set (n=220). The clinical and US characteristics of the patients are summarized in **Table 1**. There were no significant differences between the training and test sets (*P*>0.05). The unweighted values of interobserver agreement determined by Cohen’s kappa statistics ranged from 0.65 to 0.94, which showed that there was good consistency among the observers.

### Critical Predictors Obtained by Logistic Regression Analysis

Based on the univariate analysis of 20 variables for the training set (US tumor size, quantity, shape, orientation, margin contour, main posterior echo, microcalcification, echogenicity pattern, CDFI, HER-2 status, US of the lymph node, aspect ratio, cortical thickness, and central hilum), 14 predictive variables were significantly associated with ALN status (*P*<0.05). Age, margin, boundary, ER status, PR status, and Ki-67 index did not correlate with lymph node status (*P*≥0.05), as summarized in **Table 2**.

**Table 2:** Critical predictors obtained by univariate logistic regression analysis.

Features	All Patients, n(%)	ALN	ALN	*P* value
		Negative, n (%)	Positive, n (%)	
Age(y)	47.00(23.00,88.00)	47.00(23.00,86.00)	46.00(25.00,88.00)	0.130
Quantity				<0.001
Single	437(83.56)	229(43.79)	208(39.77)	
Multiple	86(16.44)	27(5.16)	59(11.28)	
Shape				0.005
Oval, round	62(11.85)	41(7.84)	21(4.01)	
Irregular	461(88.15)	215(41.11)	246(47.04)	
Orientation				<0.001
Parallel	333(63.67)	195(37.29)	138(26.38)	
Not parallel	190(36.33)	61(11.66)	129(24.67)	
Margin contour				0.044
Smooth	36(6.88)	23(4.40)	13(2.48)	
Lobulate	84(16.06)	44(8.41)	40(7.65)	
Angular, Spiculate	403(77.06)	189(36.14)	214(40.92)	
Margin				0.849
Circumscribed	40(7.65)	19(3.63)	21(4.02)	
Not circumscribed	483(92.35)	237(45.32)	246(47.03)	
Boundary				0.828
Abrupt interface	261(49.90)	129(24.67)	132(25.23)	
Echogenic halo	262(50.10)	127(24.28)	135(25.82)	
Main posterior echo				0.002
Enhancement	9(1.72)	7(1.34)	2(0.38)	
Indifferent	294(56.21)	157(30.01)	137(26.20)	
Shadowing	220(42.07)	92(17.59)	128(24.48)	
Microcalcification				<0.001
Absent	228(43.59)	134(25.62)	94(17.97)	
Preserved	295(56.41)	122(23.33)	173(33.08)	
Echogenicity pattern				0.030
Other echo	17(3.25)	13(2.49)	4(0.76)	
Hypoechoic	506(96.75)	243(46.46)	263(50.29)	
CDFI				<0.001
Adler 0,1	192(36.71)	122(23.32)	70(13.39)	
Adler 2,3	331(63.29)	134(25.62)	197(37.67)	
US tumour size(mm)	22,00(5.00,140.00)	19.00(5.00,77.00)	27.00(7.00,140.00)	<0.001
ER				0.668
Negative	171(32.70)	86(16.45)	85(16.25)	
Positive	352(67.30)	170(32.50)	182(34.80)	
PR				0.211
Negative	229(43.79)	105(20.08)	124(23.71)	
Positive	294(56.21)	151(28.87)	143(27.34)	
HER-2				0.002
Negative	379(72.47)	205(39.20)	174(33.27)	
Positive	144(27.53)	51(9.75)	93(17.78)	
Ki-67				0.121
Negative	159(30.40)	86(16.44)	73(13.96)	
Positive	364(69.60)	170(32.50)	194(37.10)	
US of lymph node				<0.001
No lymph nodes found	214(40.92)	165(31.55)	49(9.37)	
Lymph nodes found	309(59.08)	91(17.40)	218(41.68)	
Aspect ratio				<0.001
No lymph nodes found	220(42.07)	165(31.55)	55(10.52)	
Aspect ratio≥2	111(21.22)	53(10.13)	58(11.09)	
Aspect ratio<2	192(36.71)	38(7.27)	154(29.44)	
Cortical thickness				<0.001
No lymph nodes found	213(40.73)	165(31.55)	48(9.18)	
Thickness<3 mm	118(22.56)	82(15.68)	36(6.88)	
Symmetric thickness≥3 mm	19(3.63)	2(0.38)	17(3.25)	
Asymmetric thickness≥3 mm	173(33.08)	3(1.34)	166(31.74)	
Central hilum				<0.001
No lymph nodes found	207(39.58)	162(30.98)	45(8.60)	
Preserved	147(28.11)	87(16.63)	60(11.48)	
Partially absent	70(13.38)	5(0.96)	65(12.42)	
Completely absent	99(18.93)	2(0.38)	97(18.55)	

Finally, the results of the PTFM are presented in **Table 3**. The following features showed a statistically significant relationship with ALN status: US tumor size, shape, orientation, CDFI, and HER-2 status. The *P* values obtained by applying the HL test were 0.214 and 0.865 in the training and test sets, respectively, which did not indicate a significant difference. The original assumption was accepted, and there was no significant difference between the predicted value and the real value.

**Table 3:** Results of the PTFM.

Features	β	S．E	Wald z value	Multivariate OR (95% CI)	*P* value
Constant	-3.80	0.59	-6.50	0.02(0.01- 0.07)	<0.001
Quantity	0.44	0.29	1.53	1.55(0.89- 2.76)	0.127
Single					
Multiple					
Shape	0.68	0.33	2.05	1.98(1.04- 3.87)	0.040
Oval, round					
Irregular					
Orientation	1.56	0.22	6.97	4.78(3.10- 7.48)	<0.001
Parallel Not parallel					
Main posterior echo	0.30	0.20	1.49	1.35(0.91-2.01)	0.136
Enhancement Indifferent Shadowing					
Microcalcification	0.32	0.21	1.55	1.38(0.92- 2.07)	0.122
Preserved Absent					
CDFI	0.58	0.22	2.62	1.79(1.16- 2.77)	0.009
Adler 0,1 Adler 2,3					
US tumour size	0.05	0.01	5.07	1.05(1.03- 1.07)	<0.001
HER-2	0.63	0.24	2.67	1.87(1.18- 2.98)	0.008
Negative Positive					

The results of the ALNFM are displayed in **Table 4**. The following features demonstrated a significant difference that correlated independently with ALN status: US tumor size, orientation, HER-2 status, US of the lymph node, cortical thickness and central hilum. The *P* value obtained by applying the HL test was 0.369 in the training set and 0.045 in the test, which did not correspond to a significant difference. The results showed that the original assumption was accepted, and there was no difference between the predicted and actual values in the model.

**Table 4:** The results of ALNFM.

Features	β	S．E	Wald	Multivariate OR (95% CI)	*P* value
Constant	-5.07	0.94	-5.39	0.01(0.001- 0.04)	<0.001
Orientation	1.68	0.29	5.83	5.34(3.07- 9.51)	<0.001
Parallel Not parallel					
Margin contour	0.43	0.27	1.62	1.54(0.93-2.66)	0.106
Smooth Lobulate					
Angular, Spiculate					
Main posterior echo	0.46	0.27	1.69	1.58(0.93-2.70)	0.091
Enhancement					
Indifferent Shadowing					
US tumour size	0.03	0.01	2.68	1.03(1.001-1.05)	0.007
HER-2	0.65	0.30	2.13	1.91(1.05- 3.46)	0.033
Negative Positive					
US of lymph node	-3.57	0.79	-4.55	0.03(0.01- 0.12)	<0.001
No lymph nodes found					
Lymph nodes found					
Aspect ratio	0.69	0.36	1.91	1.99(0.99- 4.09)	0.057
No lymph nodes found					
Aspect ratio≥2					
Aspect ratio<2					
Cortical thickness	1.61	0.27	5.87	4.99(3.01- 8.89)	<0.001
No lymph nodes found					
Thickness<3 mm					
Symmetric thickness≥3 mm					
Asymmetric thickness≥3 mm					
Central hilum	1.18	0.39	3.05	3.25(1.58-7.28)	0.002
No lymph nodes found					
Preserved					
Partially absent					
Completely absent					

### Nomograms for Predicting ALN Status

**Figure 2:**
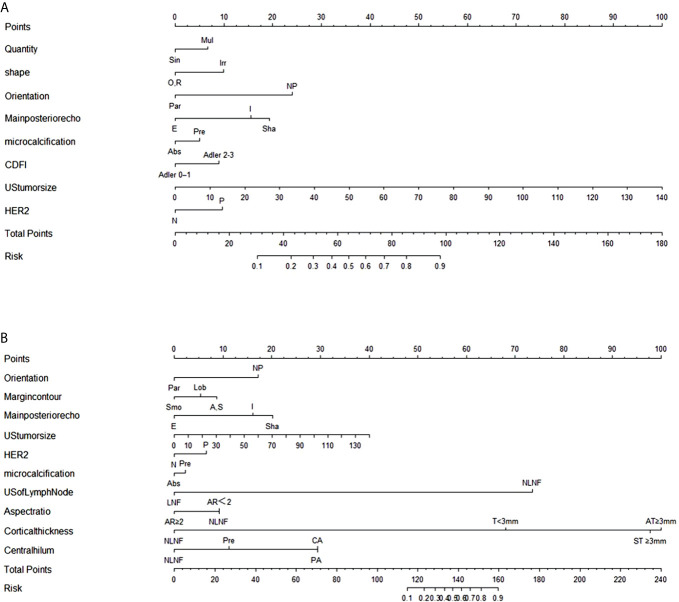
**(A)** The PTFM nomogram for predicting ALN status in the training set. **(B)** The ALNFM nomogram for predicting ALN status in the training set. Sin, Single; Mul, Multiple; O, Oval; R, round; Irr, Irregular; Par, Parallel; NP, Not parallel; Smo, Smooth; Lob, Lobulate; A, Angular; S, Spiculate; E, Enhancement; I, Indifferent; Sha, Shadowing; Abs, Absent; Pre, Preserved; N, Negative; P, Positive; NLNF, No lymph nodes found; LNF, Lymph nodes found; AR, Aspect ratio; T, Thickness; ST, Symmetric thickness; AT, Asymmetric thickness; PA, Partially absent; CA, Completely absent.

**Figure 3:**
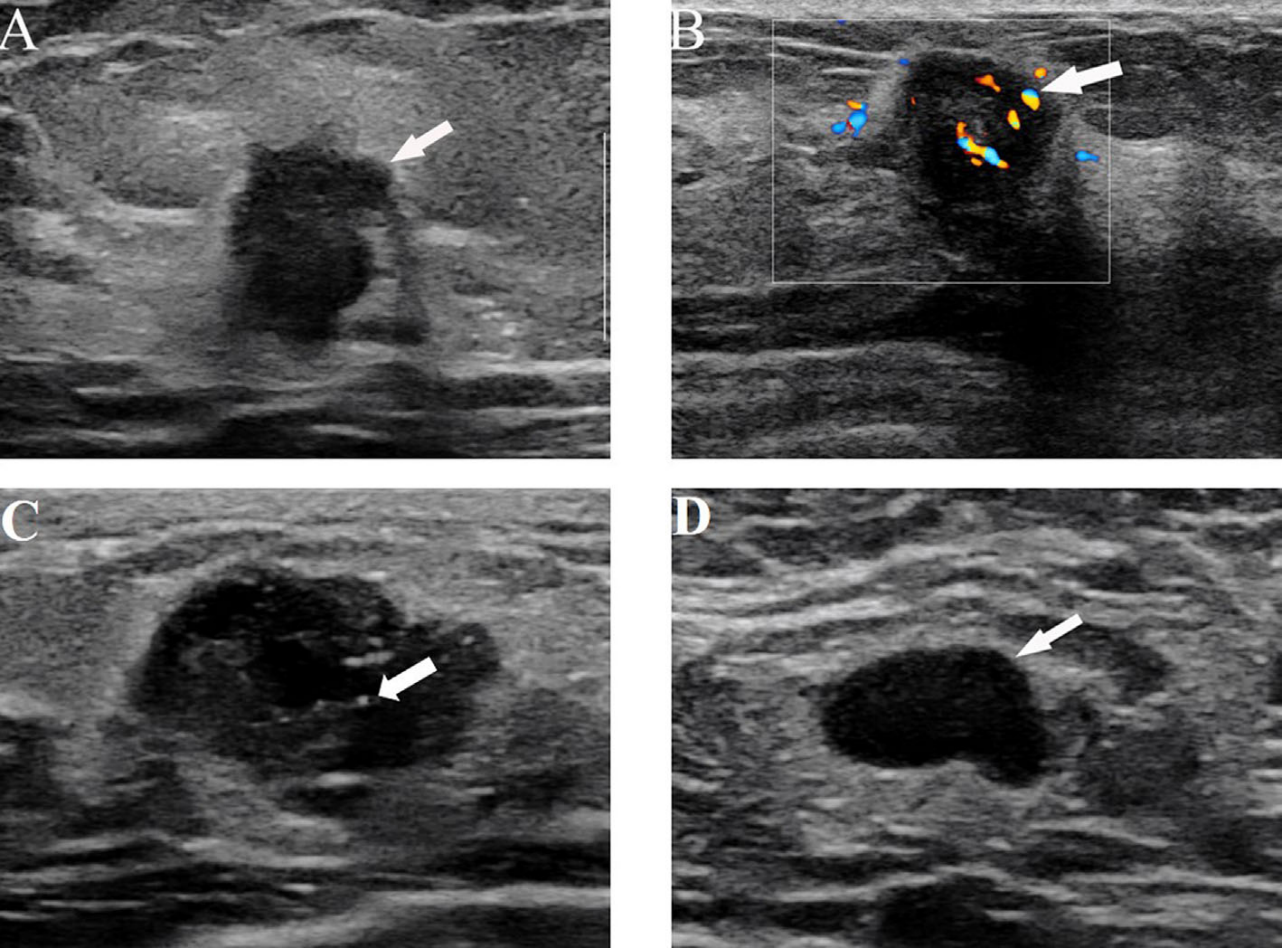
**(A)** US image from a 64-year-old woman with ALN metastasis showing the irregular shape and nonparallel growth of the tumor. **(B)** US images from a 38-year-old woman with ALN metastasis showing rich blood flow signals with the application of color Doppler flow. **(C)** US images in a 40-year-old woman with ALN metastasis, which shows microcalcification and an irregular shape. **(D)** US images from a 62-year-old woman with ALN metastasis showing the absence of a central hilum.

The stepwise regression analyses were designed to facilitate the generation of two nomograms for predicting ALN status (**Figure 2**). Irregular shape (**Figure 3A**), multiple tumors and higher CDFI classification (**Figure 3B**) were greatly associated with ALN metastasis in the PTFM nomogram. In both nomograms, nonparallel orientation growth (**Figure 3A**), main posterior shadowing, larger tumor size, microcalcification (**Figure 3C**), and HER-2 gene overexpression correlated highly positively with ALN status. In addition, a tumor margin contour that was angular or spiculate, no lymph nodes found, aspect ratio<2, thickened lymph node cortex, and absent central hilum (**Figure 3D**) correlated highly positively with ALN metastasis in the ALNFM.

### Reliability and Validation of the Nomograms

**Figure 4:**
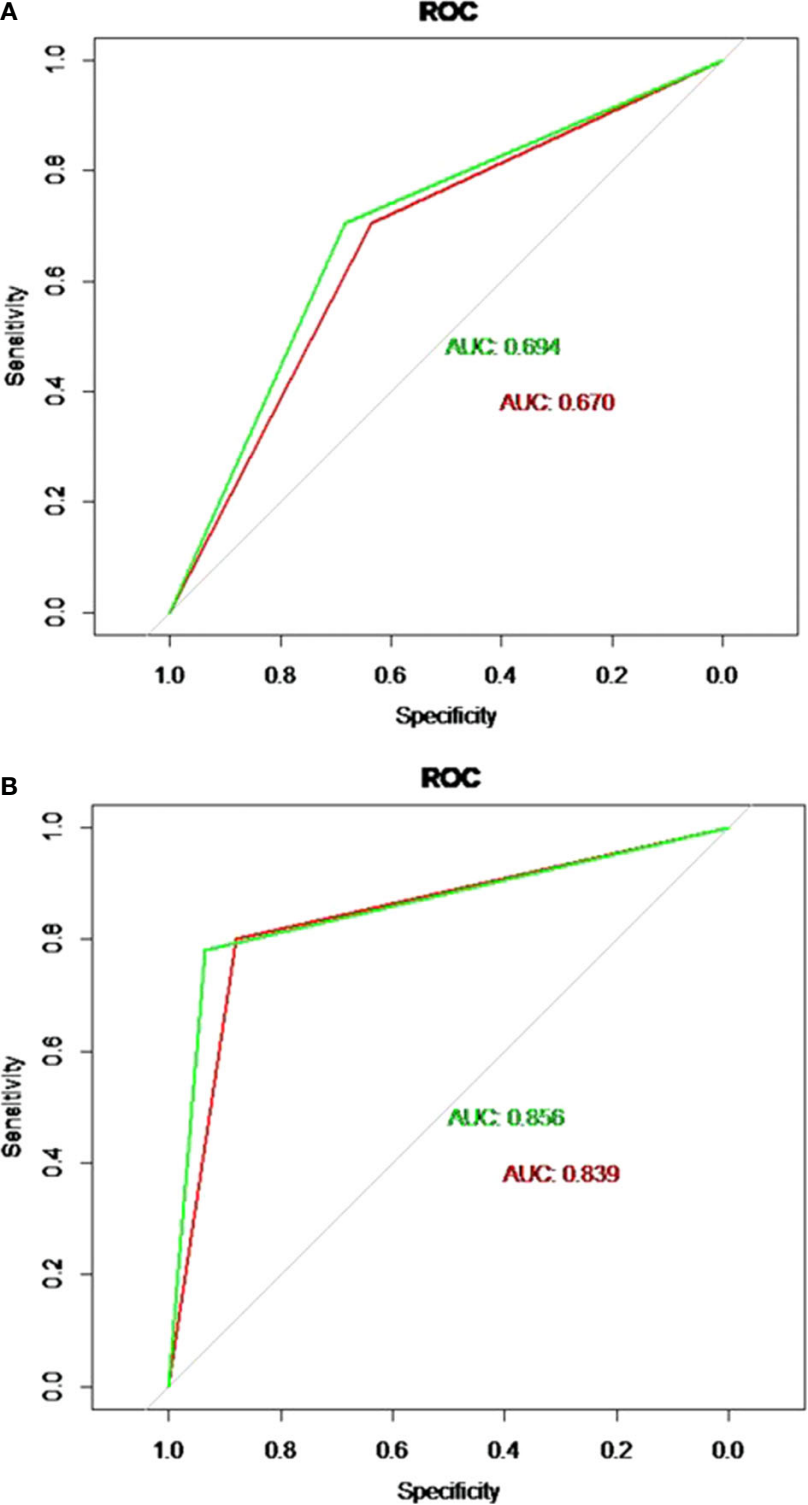
**(A)** ROC curves of the PTFM in the training set (green curve) and test set (red curve). **(B)** ROC curves of the ALNFM in the training set (green curve) and test set (red curve).

**Figure 5:**
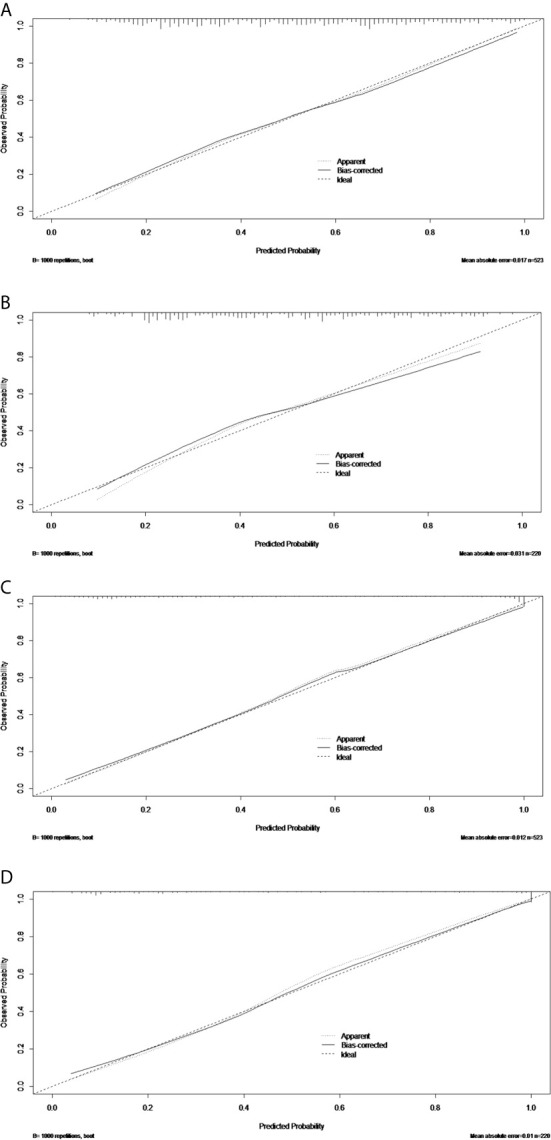
The calibration curves of the PTFM nomogram in the training set **(A)** and test set **(B)**. The calibration curves of the ALNFM nomogram in the training set **(C)** and test set **(D)**. The 45-degree diagonal line demonstrates perfect matching between the nomogram-predicted probabilities (X-axis) and the true value (Y-axis). The distances were closer between the two lines, and the prediction accuracies of the ALNFM nomogram were higher than those of the PTFM.

The discriminatory performance of the nomograms for predicting ALN status was evaluated by ROC curves. The sensitivity (Sen), specificity (Spe), and accuracy (Acu) of the PTFM were 0.70, 0.68 and 0.69, respectively, in the training set, and 0.70, 0.64 and 0.67, respectively, in the test set. The AUC values of the PTFM in the training set and the test set were 0.69 (95% CI, 0.65–0.73) and 0.670 (95% CI, 0.61–0.73), respectively (**Figure 4A**). Moreover, internal and external verification were performed using a bootstrapping method with 1000 resamples in the training and test sets. After bias correction, the AUC of the PTFM was 0.67 (−0.025) and 0.67 (−0.001) in the training set and the test set, respectively. Calibration using a bootstrapping method with 1000 resamples was also estimated graphically in the training set (**Figure 5A**) and test set (**Figure 5B**).

The Sen, Spe and Acu of the ALNFM were 0.78, 0.93 and 0.86, respectively, in the training set, and 0.80, 0.88 and 0.84, respectively, in the test set. The consistent AUCs of the ALNFM in the training set the test set were 0.86 (95% CI, 0.83–0.89) and 0.84 (95% CI, 0.79–0.89), respectively (**Figure 4B**). Moreover, internal and external validations were performed using a bootstrapping method with 1000 resamples in the training and test sets. After bias correction, the AUC of the ALNFM was 0.85 (−0.003) and 0.81 (−0.034) in the training and test sets, respectively. Calibration using a bootstrapping method with 1000 resamples was also estimated graphically in the training set (**Figure 5C**) and test set (**Figure 5D**). The distance between the two lines of the ALNFM was less than that of the PTFM. Compared with the PTFM, the ALNFM achieved the best performance in both the training and test sets (*P*<0.001).

### Prediction Performance of the ALNFM Using US Tumor Size

ROC curve analysis was used to select the cut-off point of tumor diameter according to ALN status, and the cut-off value was 21.5mm (**Supplementary Figure 1**). The cut-off values of the Sen and Spe were assessed by application of the ALNFM. When the US tumor size was ≤21.5 mm, the Spe was 0.96 and 0.92 in the training and test sets, respectively. Moreover, with the decrease in US tumor size, the Spe increased significantly. When the US tumor size was greater than 21.5mm, the Sen was 0.85 in the training set and 0.87 in the test set. Furthermore, when the US tumor size increased, the Sen also increased significantly. Our further research showed that when the US tumor size was larger than 35mm, the Sen was 0.90 in the training set and 0.93 in the test set (**Supplementary Table 1**).

### Clinical Value of Evaluation of the Nomograms

**Figure 6:**
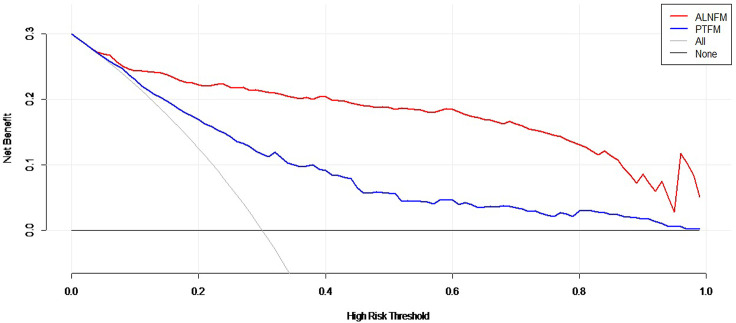
Decision curve analysis of the two nomograms. The net benefit is shown by the y-axis. The advantages minus disadvantages are equal to the net benefits. When the risk threshold probability is between 0.07 and 0.99, the ALNFM nomogram (red curve) showed more benefit for patients for predicting ALN status than the PTFM nomogram or the treat-all and treat-none schemes.

Decision curve analysis was used to assess the clinical utility of the two prediction nomograms (**Figure 6**). It showed that there was an ideal high net benefit. The nomogram constructed with the ALNFM showed the most clinical benefits for predicting ALN status when the risk threshold probability for patients ranged from 0.07 to 0.99. The nomogram constructed with the ALNFM showed more benefit than the nomogram constructed with the PTFM. Therefore, our nomogram constructed with the ALNFM performed better.

## Discussion

ALN status has been considered an important factor for determining treatment and prognosis of patients (25, 26). Currently, the treatment of breast cancer patients is developing toward to a less invasive treatment of the ALNs, involving less pain and close follow-up (3). If the ALN status can be effectively predicted preoperatively, the surgical plan can be optimized and unnecessary invasive examination of the ALNs can be omitted to reduce trauma. US imaging for diagnosis of ALN status has become a concern in recent years, but this issue is still in the early stages of research, and there is no uniform diagnostic standard. Few studies have been performed on the US features of breast masses to predict ALN status; therefore, we attempted to use easily acquired clinical features and preoperative US features of breast masses and ALNs to predict ALN status and develop forecasting nomograms. A profound study of new methods of traditional US has been performed, which should be brought to the attention of clinical doctors. Further research on traditional US still has a broad application prospect and high application value with the current rapid development of US technology.

To compare the performance of the prediction model of clinical features and US features of primary breast tumors with the model combined with US features of ALNs, we established two nomograms in this study. We found that the ALNFM was more significant than the PTFM (*p*<0.001). The PTFM nomogram included the characteristics of breast tumors and did not include the characteristics of the ALNs. This nomogram used an indirect method, while the ALNFM used a more direct method. Thus, the PTFM nomogram was less significant than the ALNFM nomogram. However, except for the association of the same features (nonparallel orientation growth, main posterior shadowing, microcalcification, larger tumor size, and HER-2 gene overexpression) with ALN metastasis, the PTFM nomogram incorporated more US features of primary breast tumors, such as multiple tumors, irregular tumors, and abundant blood supply. Sonographers should be reminded to pay special attention to the status of ALNs when they find the above features during a scan.

The performance of the ALNFM (AUC: 0.86 and 0.84 in the training set and the test set, respectively) was not only better than that of the PTFM, but it was also better than that of other previous methods, including dynamic contrast-enhanced MRI, clinicopathological features and the previous Memorial Sloan-Kettering Cancer Center (MSKCC) model, and the AUCs of previous studies ranged from 0.67 to 0.82 (12, 13, 27). The MSKCC model has been verified in other countries in succession, and the AUC ranged from 0.63 to 0.78 (28–30). Meanwhile, our results were also reliable and objective. First, there was little bias in the AUC values in the internal and external verification. The calibration curves were closer to the 45 degree diagonal line, and the prediction accuracies were higher. Second, decision curve analysis showed a higher clinical application value and better clinical practicability. The results of some studies were also consistent with those of our study, with AUCs ranging from 0.85 to 0.86 (31, 32). The nomogram showed that nonparallel orientation growth, a tumor margin contour that was angular or spiculate, main posterior shadowing, larger tumor size, microcalcification, aspect ratio<2, thickened lymph node cortex, absent central hilum, and HER-2 gene overexpression were likely to be associated with ALN metastasis. Therefore, if a breast tumor is detected by preoperative US and accompanied by any of the above conditions, especially larger tumor size, thickened lymph node cortex, or absent central hilum, metastasis of the ALNs could be predicted, and preoperative core needle biopsy, SLNB or ALND should be performed. The Spe of the ALNFM was higher (0.93 and 0.88 in the training and test sets, respectively). Moreover, if the breast tumor was not accompanied by any of the above conditions and the ALNs were predicted to be negative, unnecessary preoperative core needle biopsy, SLNB or ALND may be avoided to a certain extent. Despite the rapid development of US technology, traditional US is still dominant and irreplaceable; at the same time, traditional US combined with clinicopathological characteristics for predicting ALN is currently the most practical and concise new method and is easier to perform and more stable than the radiomics method or elastography (33, 34). In particular, US is a noninvasive and routine method for evaluating breast cancer and can be widely used, even in some underdeveloped areas.

After the best model was selected, we performed in-depth research on the clinical significance of the ALNFM nomogram developed from traditional US and further explored the critical US features associated with ALN metastasis. This study showed that the US tumor size was associated with ALN metastasis, and the results were in agreement with those of a previous study (35, 36). Furthermore, the threshold was 21.5 mm, which was surprisingly similar to the endpoint value (20 mm) of T1 and T2 and this result was consistent with those of previous studies that showed that a tumor size greater than 20 mm was an important independent risk factor for ALN metastasis (37). The study showed that when the US tumor size was ≤21.5 mm, the Spe was ideal(with values of 0.96 and 0.92 in the training and test sets, respectively); when the US tumor size was greater than 21.5 mm, the Sen was very high (0.85 and 0.87 in the training and test sets, respectively); when the US tumor size was larger than 35 mm, the Sen was ideal(with values of 0.90 and 0.93 in the training and test sets, respectively), and previous studies believe that it was ideal when the false negative rate (1-sensitivity) of a model was less than 10% (38). Thus, when the US tumor size was ≤21.5 mm, the ALN status was predicted to be negative, SLNB or core needle biopsy may not be recommend for surgical staging, and close follow-up should be recommend to some small tumors. when the US tumor size was >21.5 and ≤35 mm, the ALN procedure was selected based on the combination of clinical and ultrasound features related to ALN metastasis. If the US tumor size was greater than 35mm and ALN is predicted to be negative, invasive ALN biopsy, sentinel lymph node biopsy and dissection may be avoided and when the ALN status was predicted to be positive, core needle biopsy, SLNB or ALND was absolutely necessary. Thus, the clinical application value of the ALNFM according to US tumor size was obviously significant.

Our study indicated that the shape, orientation and CDFI were markedly independently associated with ALN metastasis. The presence of multiple tumors, a margin contour that was angular or spiculate, posterior echo shadowing, hypoechoic and microcalcification were obviously associated with ALN metastasis based on univariate logistic regressions analysis. The results were consistent with those of previous studies (22, 27). We thought that the results might be related to the biological behavior of tumors; the more likely a tumor is to metastasize, the faster it grows, the larger it grows, the greater the number of tumors, the more unbalanced it is, the more angular or spiculate it is, the more irregular its shape is, the more likely it is to grow in a nonparallel orientation, and the more abundant the blood supply is. Furthermore, because it is growing too fast, the nutrition is often in poor supply, so the tumor is more prone to microcalcification and more likely to be accompanied by posterior echo shadowing (39). The study also showed that aspect ratio<2, thickened lymph node cortex and absent central hilum were independently positively associated with ALN metastasis. Clinical studies have confirmed that cancer cells transfer to the lymph nodes and destroy the cortex, medulla and central hilum, leading to enlarged ALNs, a thickened cortex, enhanced lateral growth, and a diminished aspect ratio (40, 41). When the ALNs were found by ultrasound, the impacts of ALN features on the predicting results will be highlighted. Our research found that even if ALNs were not found by ultrasound, there were 22.90% (49/214) ALN metastases. When the ALNs were not found by ultrasound, the impacts of the ALN features on the predicting results were absent, therefore, the impacts of No lymph nodes found on the predicting results will be highlighted. These may explain why no lymph nodes found correlated highly positively with ALN metastasis. This study also confirmed that the molecular phenotype was related to ALN status, and patients with HER-2 positivity had a higher metastasis risk, which was consistent with the results of other experts who found that HER-2 gene overexpression in breast cancer was associated with a higher degree of malignancy and an increased likelihood of metastasis (24).

Regardless of the development of US technology in the future, the simple and easy-to-use traditional US always has broad application prospects in predicting ALN status. Compared with difficult, challenging, and expensive methods, US has great importance in clinical practice; moreover, it had greater guiding significance in the procedure of ALNs than previous studies and postoperative methods. Therefore, irrespective of how many models there are, application value is the most important determinant.

## Data Availability Statement

The raw data supporting the conclusions of this article will be made available by the authors, without undue reservation.

## Ethics Statement

The studies involving human participants were reviewed and approved by the Ethics Committee of the Second Affiliated Hospital of Harbin Medical University. The patients/participants provided their written informed consent to participate in this study. Written informed consent was obtained from the individual(s) for the publication of any potentially identifiable images or data included in this article.

## Author Contributions

DL, JT, and XL designed and guided the study. DL and LZ analyzed the imaging data. TW collected the ultrasound images. HC, ZL, PS, and PT collected the clinical data. DL, YL, and XL performed the statistical analysis. DL wrote the manuscript. JT revised the manuscript. All authors contributed to the article and approved the submitted version.

## Funding

This work was supported by the National Natural Science Foundation of China under grants 81630048, 81701705, 81974265, and 81901761; postdoctoral Funding of Heilongjiang Province under grant LBH-Z17174; and scientific research project of Heilongjiang Health Committee no. 2019-050.

## Conflict of Interest

The authors declare that the research was conducted in the absence of any commercial or financial relationships that could be construed as a potential conflict of interest.

## Supplementary Material

Supplementary Figure 1 ROC curve of tumor diameter with ALN status.
