# Supplementary figures and images for: Nomograms for Predicting Axillary Lymph Node Status Reconciled With Preoperative Breast Ultrasound Images
Source: Front Oncol. 2021 Apr 7;11:567648. doi: 10.3389/fonc.2021.567648 (PMC8058421; doi:10.3389/fonc.2021.567648)

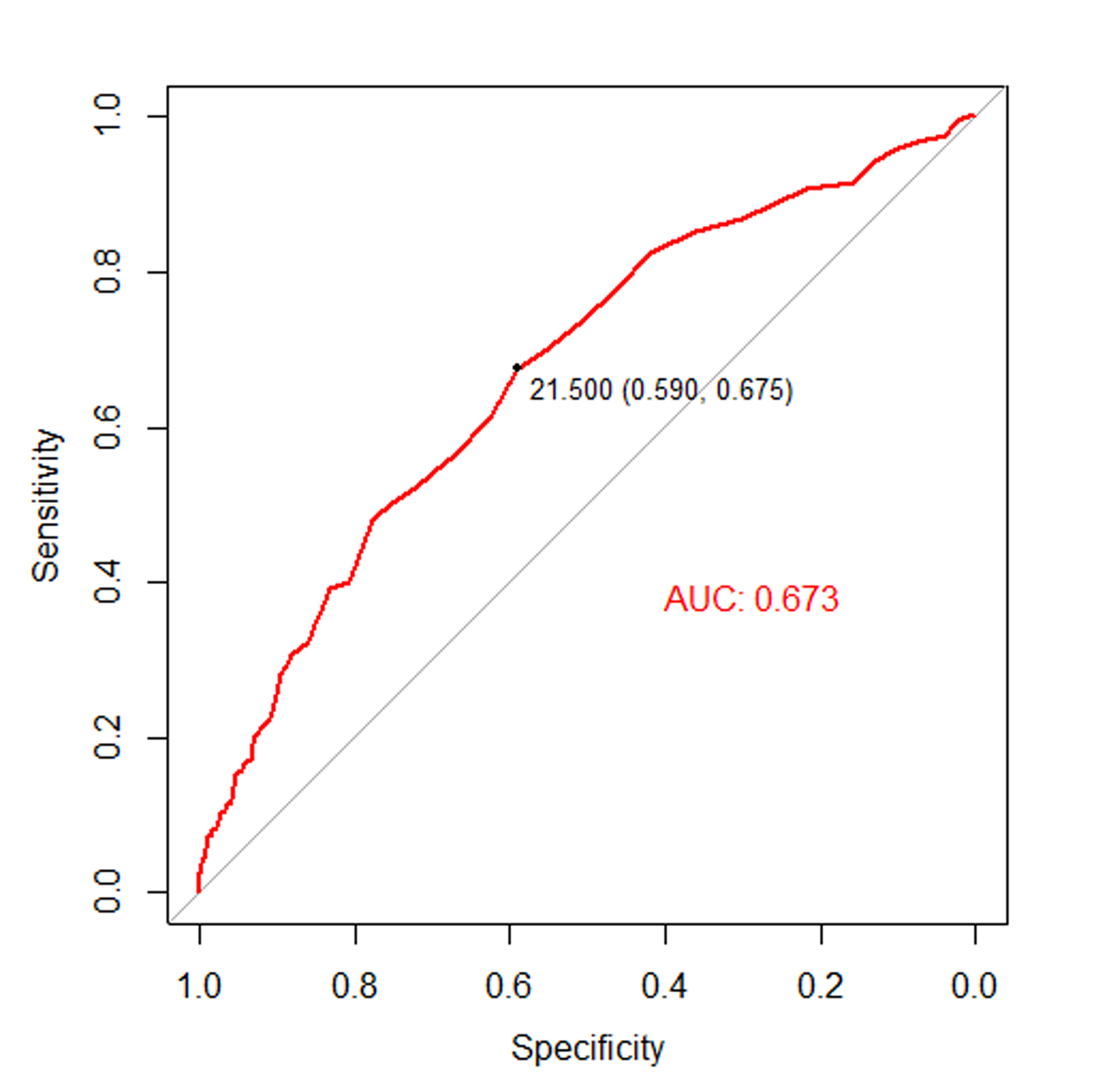

Supplement: Supplementary Figure 1 — ROC curve of tumor diameter with ALN status. [file Image_1.jpg]
