# Supplementary material for: Nomograms for Predicting Axillary Lymph Node Status Reconciled With Preoperative Breast Ultrasound Images
Source: Front Oncol. 2021 Apr 7;11:567648. doi: 10.3389/fonc.2021.567648 (PMC8058421; doi:10.3389/fonc.2021.567648)
Supplement: Supplementary file 2 [file Table_1.docx]

**Supplementary Table 1** **|** Prediction performance of ALNFM according to tumour size on US.

|  | Training set(n=523) | | |  | Test set(n=220) | | |
| --- | --- | --- | --- | --- | --- | --- | --- |
| US tumor size | NO.of patients, n(%) | Sen | Spe |  | No.of patients, n(%) | Sen | Spe |
| ≤21.5mm | 241(46.08) | 0.63 | 0.96 |  | 99(45.00) | 0.66 | 0.92 |
| >21.5mm | 282(53.92) | 0.85 | 0.90 |  | 121(55.00) | 0.87 | 0.82 |
| >35mm | 109(20.84) | 0.90 | 0.82 |  | 45(20.45) | 0.93 | 0.73 |
